# Proton pump inhibitor alters Th17/Treg balance and induces gut dysbiosis suppressing contact hypersensitivity reaction in mice

**DOI:** 10.3389/fimmu.2024.1390025

**Published:** 2024-08-23

**Authors:** Anna Strzępa, Katarzyna Marcińska, Aneta Kiecka, Monika Majewska-Szczepanik, Marian Szczepanik

**Affiliations:** ^1^ Faculty of Health Sciences, Institute of Physiotherapy, Jagiellonian University Medical College, Cracow, Poland; ^2^ Department of Medical Physiology, Faculty of Health Sciences, Institute of Physiotherapy, Jagiellonian University Medical College, Cracow, Poland

**Keywords:** proton pump inhibitors, omeprazole, contact hypersensitivity, gut microbiota, dysbiosis, immunomodulation

## Abstract

Proton pump inhibitors (PPIs), such as omeprazole, are the most commonly prescribed drugs. Treatment with PPIs alters gut microbiota composition and reduces the production of reactive oxygen (ROS) and proinflammatory IL-1β, IL-6, and TNF-α cytokines. Here, using the T cell-dependent contact hypersensitivity (CHS) response, an animal model of allergic contact dermatitis (ACD) that affects up to 30% of the population, we demonstrated that a two-week omeprazole treatment suppresses the development of CHS. Omeprazole treatment before CHS induction, reduced inflammatory response in ears measured by ear swelling, ear biopsy weight, MPO activity, and proinflammatory cytokine production. These changes were associated with reduced frequency of TCRαβ^+^ CD4^+^ IL-17A^+^ and TCRαβ^+^ CD8^+^ IL-17A^+^ T cells and increased frequency of TCRαβ^+^ CD4^+^ CD25^+^ FoxP3^+^ Treg, and TCRαβ^+^ CD4+ IL-10+ Tr1 cells in peripheral lymphoid organs. Omeprazole treatment decreased the production of ROS, TNF-α, and IL-6, which supported Th17 cell induction, and increased the frequency of *Clostridium* cluster XIVab and *Lactobacillus*, implicated in Treg cell induction. The fecal microbiota transplantation (FMT) experiment confirmed the role of omeprazole-induced changes in gut microbiota profile in CHS suppression. Our data suggests that omeprazole ameliorates inflammatory response mediated by T-cells.

## 1 Introduction

Proton pump inhibitors (PPIs), such as omeprazole, are among the top 10 most commonly prescribed drugs in the US (1), used for the treatment of acid-related digestive system diseases, such as esophagitis, gastroesophageal reflux disease, peptic ulcer disease among few. Due to the excellent safety profile of PPIs, long-term, even lifelong use is not uncommon (2). However, PPIs are one of the drugs with the strongest potential to modulate gut microbiota composition (3).

PPIs upon activation posed by acid-induced cleavage within acidic secretory canaliculi, inhibit H^+^/K^+^ ATPase of gastric parietal cells blocking acid production (1). Increased gastric pH impedes vitamin B12, iron, and magnesium absorption and alters intestinal microbiota composition (4, 5) by enabling bacteria translocation across the gastric barrier, changing digestive content and its distribution across the digestive system, influencing hormone balance (6), as well directly impacting bacterial H^+^/K^+^ ATPase of several microorganisms (7). PPIs application decreases the abundance of intestinal bacteria, simultaneously increasing the number of oral bacteria in the intestines (47). Although PPIs-induced gut microbiota alteration is implicated in increased susceptibility to *Clostridium difficile–*associated disease and promotes intestinal bacterial overgrowth (8) several studies showed anti-inflammatory properties of PPIs (9).

*In vitro* studies showed that PPIs inhibit reactive oxygen production by up-regulating SOD and catalase, reduce the production of proinflammatory TNF-α, IL-1β, and IL-6 cytokines (10, 11), and the production of Th2-type cytokines IL-4 and IL-13 (12). Furthermore, PPIs reduce neutrophil degranulation and chemotaxis (13), which could be a consequence of reduced expression of endothelial cell intercellular adhesion molecule-1 (ICAM-1) and vascular adhesion molecule-1 (VCAM-1) after PPIs treatment (14, 49). However, the anti-inflammatory properties of PPIs did not manage to ameliorate clinical scores in experimental autoimmune encephalomyelitis (EAE) (15), despite changes in gut microbiota profile (15). Contrary omeprazole administration effectively ameliorated ovalbumin-induced asthma, what may be related to reduce Th2-type cytokines production (12).

Contact hypersensitivity (CHS) is an animal model of allergic contact dermatitis (ACD), a disease that arises from exposure to low-molecular-weight substances and ions and affects up to 15% of the general population and 30% of working people (16). CHS is a type IV hypersensitivity reaction mediated by T cells, the mechanism that drives many autoimmune disorders (17).

CHS consists of inductive and effector phases. During induction, skin exposure to hapten solution results in self-protein modification by hapten and activation of innate immune response in the skin. Proinflammatory IL-1β and TNF-α activate dermal dendritic cells (DDC), which pick up haptenized-self protein and migrate to draining lymph nodes, where the hapten-specific CD4^+^ and CD8^+^ Th1 cells and Th17 cells are induced (18–20). The effector phase is induced after 4-5 days by applying a hapten solution somewhere else in the body. Migration of hapten-specific T cells to the site of subsequent exposure, along neutrophils and macrophages, driven by expression of adhesion molecules on endothelial membranes, results in skin inflammation (skin swelling) that peaks 24 to 48 h after hapten challenge.

The magnitude of CHS response is controlled during induction by the levels of proinflammatory IL-1β and TNF-α and anti-inflammatory IL-10 during immunization (18) and the proinflammatory potential of dendritic cells (50) dependent on the production of reactive oxygen species (ROS) and proinflammatory cytokines (21). Various populations of regulatory cells were implicated in the modulation of induction and elicitation of CHS (17), and their induction is influenced by gut microbiota composition (17).

Given the common long-term use of PPIs and their potential to modulate gut microbiota composition—a well-known factor in regulating systemic immune response—our study aimed to investigate the consequences of prolonged omeprazole use on the development of T cell-dependent immune responses.

## 2 Materials and methods

### 2.1 Mice

Female and male C57BL/6 mice 6-10 weeks old were from the breeding unit of the Department of Biomedical Sciences, Jagiellonian University, College of Medicine. Animals were kept under pathogen-free conditions in individually ventilated cages using the Aero-Mouse IVC Green Line system (Tecniplast S.p.A., Buguggiate, Italy) and fed autoclaved food and water ad libitum. The experiments were approved by the 1^st^ Ethical Committee for Animal Experimentation in Krakow (approval no. LKE 256/2019).

### 2.2 Reagents

Trinitrochlorobenzene (TNP-CL, synonym- TNCB) (Chemica Alta, Edmonton, Canada); hexadecyltrimethylammonium bromide, o-dianisidine dihydrochloride, and hydrogen peroxide, lysozyme, magnesium chloride, JumpStart™ Taq ReadyMix™ for qPCR, o-dianisidine dihydrochloride, sodium dodecyl sulfate and Tris-EDTA, RPMI 1640, fecal clef serum (FCS), were purchesed from Sigma (St. Louis, MO, USA). Silica beads were from BioSpec Products (USA); proteinase K was from Roche Diagnostics (Germany); 3,3’,5,5’-tetramethylbenzidine (TMB) (BD Biosciences, San Jose, CA); omeprazole (Zentiva, Czech Republic).

### 2.3 Induction and elicitation of contact hypersensitivity *in vivo*

**Scheme 1:**
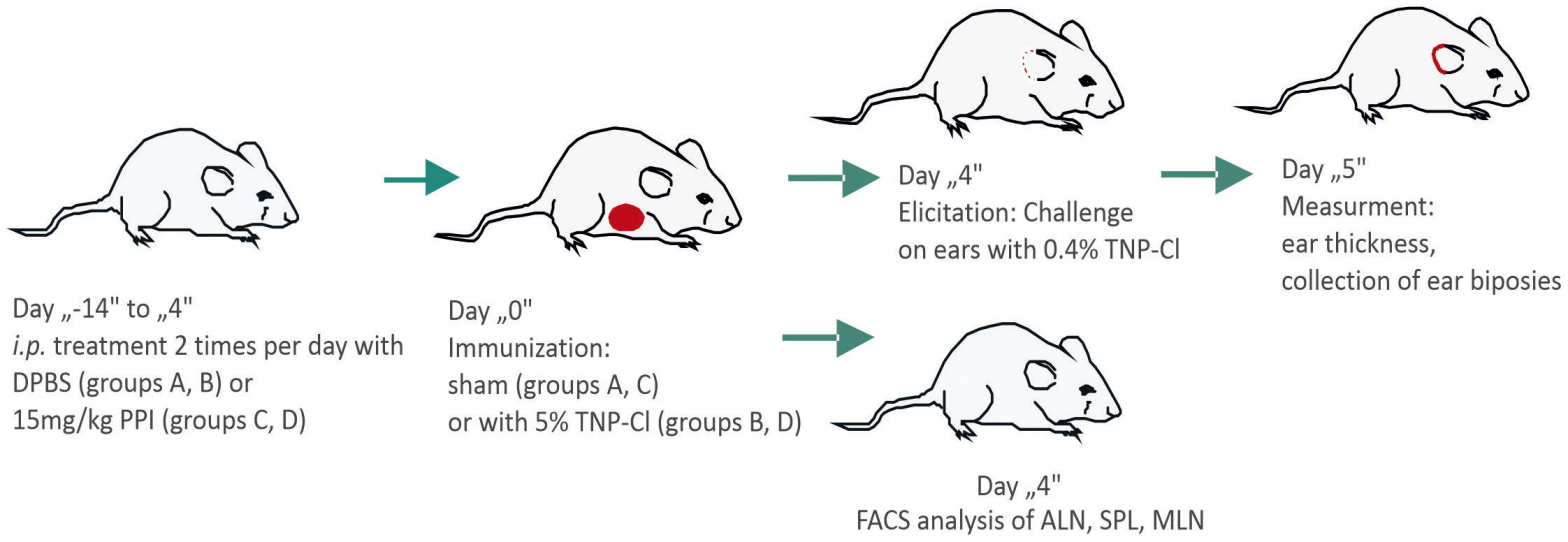
Scheme of the experiment. Mice were divided into four groups: A (Ve-), B (Ve+), C (Ve- PPI), and D (Ve+ PPI). Groups C and D were treated intraperitoneally with omeprazole (PPI) in 100 µl DPBS (15 mg/kg) twice daily for 14 days prior to immunization and continued for 4 days until ear challenge. Groups A and B received DPBS alone following the same schedule. Groups B and D were immunized with 150 µl of 5% TNP-Cl in an acetone-ethanol mixture applied to the shaved abdomen, while groups A and C received only the acetone-ethanol mixture (sham-sensitized). Four days after immunization, all mice were challenged on both ears with 10 µl of 0.4% TNP-Cl in an olive oil-acetone mixture. Ear thickness was measured before challenge and 24 hours post-challenge using a micrometer (Mitutoyo, Tokyo, Japan) by an observer blinded to the experimental groups. Ear biopsies were collected for MPO and cytokine analysis. Alternatively, four days after immunization, the mice were sacrificed, and the axillary lymph nodes (ALN), spleen (SPL), and mesenteric lymph nodes (MLN) were collected and stained with fluorochrome-conjugated antibodies for FACS analysis.

Mice were immunized by application of 150µl of 5% trinitrochlorobenzene (TNP-Cl) in an acetone-ethanol mixture (1:3) to the shaved abdomen (positive group: Ve+). Control mice were sham-immunized by applying an acetone-ethanol mixture (1:3) alone (negative control group: Ve-). Four days later, all the mice were challenged on both ears with 10µl of 0.4% TNP-CL in the olive oil-acetone mixture (1:1). Ear thickness was measured using a micrometer (Mitutoyo, Tokyo, Japan) by an observer blinded to the experimental groups. The calculation of ear thickness was performed as follows: (Ear thickness [μm] 24 hours after challenge) - (Ear thickness [μm] before challenge). The negative control group consisted of littermate sham-sensitized animals that underwent similar challenges (**Scheme 1**).

### 2.4 Treatment with omeprazole

Mice were treated intraperitoneally (*i.p.*) with omeprazole (PPI) in 100 µl DPBS (15mg/kg) or DPBS alone twice daily for 14 days prior to TNP-CL or sham immunization and then for the consecutive 4 days until the ear challenge.

The groups are as follows: A (Ve-): Negative control group, which was treated with DPBS daily and sham-immunized. B (Ve+): Positive control group, which was treated with DPBS daily and immunized with hapten. C (Ve- PPI): Negative control group, which was treated daily with PPI and sham-immunized. D (Ve+ PPI): Study group, which was treated daily with PPI and immunized with hapten (**Scheme 1**).

Ear swelling was further confirmed by measuring cytokine concentrations in ear biopsies. In brief, a 6-mm diameter ear punch was collected and homogenized in T-PER™ Tissue Protein Extraction Reagent (Thermo Scientific) containing a mixture of proteinase inhibitors using a tissue homogenizer. The homogenates were then centrifuged (10,000 x g, 10 minutes, 4°C), and the supernatants were tested for cytokine concentration by ELISA, using sets purchased from BD Pharmingen, San Diego, CA. This method was also employed to process 1 cm-long small intestine biopsies.

### 2.5 Myeloperoxidase assay

The MPO assay was used to indirectly quantify neutrophil infiltration into the inflamed tissues, as described previously. 24 h post challenge and a 6-mm diameter ear punch was collected and homogenized in 0.5% hexadecyltrimethylammonium bromide pH = 6.0 (50 mg of tissue/ml). The homogenates were frozen – thawed 3 times, centrifuged at 40,000 g, 0.1 ml aliquots were mixed with 2.9 ml phosphate buffer (pH = 6.0) containing 0.167 mg/ml of o-dianisidine dihydrochloride and 5x10^-4^% H_2_O_2_ and incubated at 25°C for 20 min. The absorbance was measured at 460 nm in 96-well flat bottom plates. MPO activity was expressed in units per protein concentration (U/mg of protein).

### 2.6 Cell culture

To characterize the change in the production of cytokines after omeprazole treatment, 3 x 10^6^ cells isolated mesenteric lymph nodes (MLNC), and auricular lymph nodes (ELNC) from mice treated with DPBS or omeprazole prior to sham- or TNP-CL-immunization were cultured in 1 ml RPMI 1640 medium supplemented with penicillin, streptomycin, NEAA, and 10% FCS in the presence of antigen (100 μg/ml TNP-Ig) in flat 24 well Falcon plates. After 48 h, the culture supernatants were collected and samples were tested for cytokine concentration by ELISA, using sets purchased from BD Pharmingen, San Diego, CA.

### 2.7 Flow cytometry

For flow cytometry analysis, ALN, SPL, MLN were collected and PP were excised from intestines of mice treated DPBS or omeprazole prior to sham- or TNP-CL-immunization. Collected tissues were grinded using glass slides and tissues fragments were removed using cell strainers. The cells were washed with PBS and 1% FCS, centrifuged (1200 rpm, 10 min, 4°C) and cell pellets were re-suspended in staining buffer.

For T cell phenotyping, anti-CD4-FITC, anti-CD8-PeCy7, anti-TCRγδ-PerCP-Cy5.5, anti-TCRβ-APC-Cy7 (BioLegend, San Diego, CA), and anti-CD25-APC mAbs were used. For DC analysis anti-MHCII-FITC, anti-CD11b-Pe-Cy7, anti-CD11c-APC-Cy7 anti-CD103-Per-Cy5.5 (BioLegend, San Diego, CA, USA) mAbs were used.

Treg cells were determined by staining with anti-FoxP3-PE mAb (BD Biosciences, San Jose, CA) using the mouse Treg staining kit (eBioscience) according to the manufacturer’s instructions.

To detect intracellular IL17A, IL-10, and IFN-γ, ICC (intracellular cytokine) staining was performed. Single-cell suspensions were cultured for 4 h with phorbol 12-myristate 13-acetate (PMA), ionomycin (Sigma Chemical Co., St Louis, MO) and Golgi Plug (eBioscience, San Diego, CA) prior to staining for T cell surface markers. Finally, the cells were fixed and permeabilized with an intracellular staining buffer kit from eBioscience Inc. (San Diego, CA, USA) prior to staining with anti-IFN-γ-APC, anti-IL-10-APC or anti-IL17A-PE mAbs, (BD Biosciences, San Jose, CA, USA).

To detect reactive oxygen species ROS-sensitive dye was used, CellROX Deep Red Reagent (Invitrogen™). Immune cells resuspended in PBS were stained with 5 μM CellROX Deep Red Reagent at 37°C in the dark for 30 min, washed in, and resuspended in PBS, followed by incubation with Fc blocker and staining for surface markers.

The stained samples were then assessed by flow cytometry using a FACSCanto II instrument (BD Biosciences), and data was analyzed using FACS DIVA software. Gating strategy provided as **Supplementary Material**.

### 2.8 Extraction of bacterial DNA from the gut content

Gut content was harvested by washing out with sterile DPBS and centrifuged. The pellet was resuspended in sterile DPBS and mixed vigorously. After centrifugation, the supernatant without the solid fraction was collected and centrifuged. The pellet was resuspended in 300 µl of Tris-EDTA. Samples were frozen and thawed several times followed by chemical disruption with lysozyme and SDS, followed by protein removal using silica beads. DNA extraction with phenol:chloroform:isoamyl alcohol (25:24:1) solution was performed (17).

### 2.9 PCR conditions

To evaluate dysbiosis after PPI treatment, RT-PCR was performed with 10.5 ng of DNA using CFX96 Touch. The detection of selected gut bacteria species and groups was based on the amplification of conserved 16S rDNA fragments. PCR conditions were as described previously (17). 16S rDNA detected by universal primers and probes, detecting all bacteria, was used as a reference gene. The expression level was evaluated by the values received from mixed DNA samples extracted from control mice and presented as ΔΔct. Values for all bacteria were subtracted from values obtained for selected bacteria (17).

### 2.10 Fecal microbiota transplantation

The experiment of “adoptive microbiota transfer” (FMT- fecal microbiota transplantation) was performed by oral gavage with the fecal material from the donors that were treated intraperitoneally with 100 µl omeprazole (15 mg/kg) or DPBS alone twice daily for 14 days. 12 h after stopping omeprazole, the lumen contents of the large intestine were harvested in 4 ml of sterile PBS, mixed vigorously, and centrifuged. The fecal supernatant (300 µl) was orally inoculated into naïve recipient mice. FMT was performed twice a week for two weeks using fresh fecal supernatant each time (17) prior to CHS induction.

### 2.11 Statistical analysis

The data with a normal distribution were analyzed using ANOVA followed by Bonferroni’s Selected Pairs of Column Comparison Test for comparisons between multiple groups. For small sample sizes and two-group comparisons, the unpaired t-test was used (22). Experiments were repeated at least twice. Data are presented as mean values ± SEM, and a p-value ≤0.05 was considered significant using GraphPad Prism (San Diego, CA).

## 3 Results

### 3.1 Treatment with proton pump inhibitor alleviates CHS in mice

**Figure 1:**
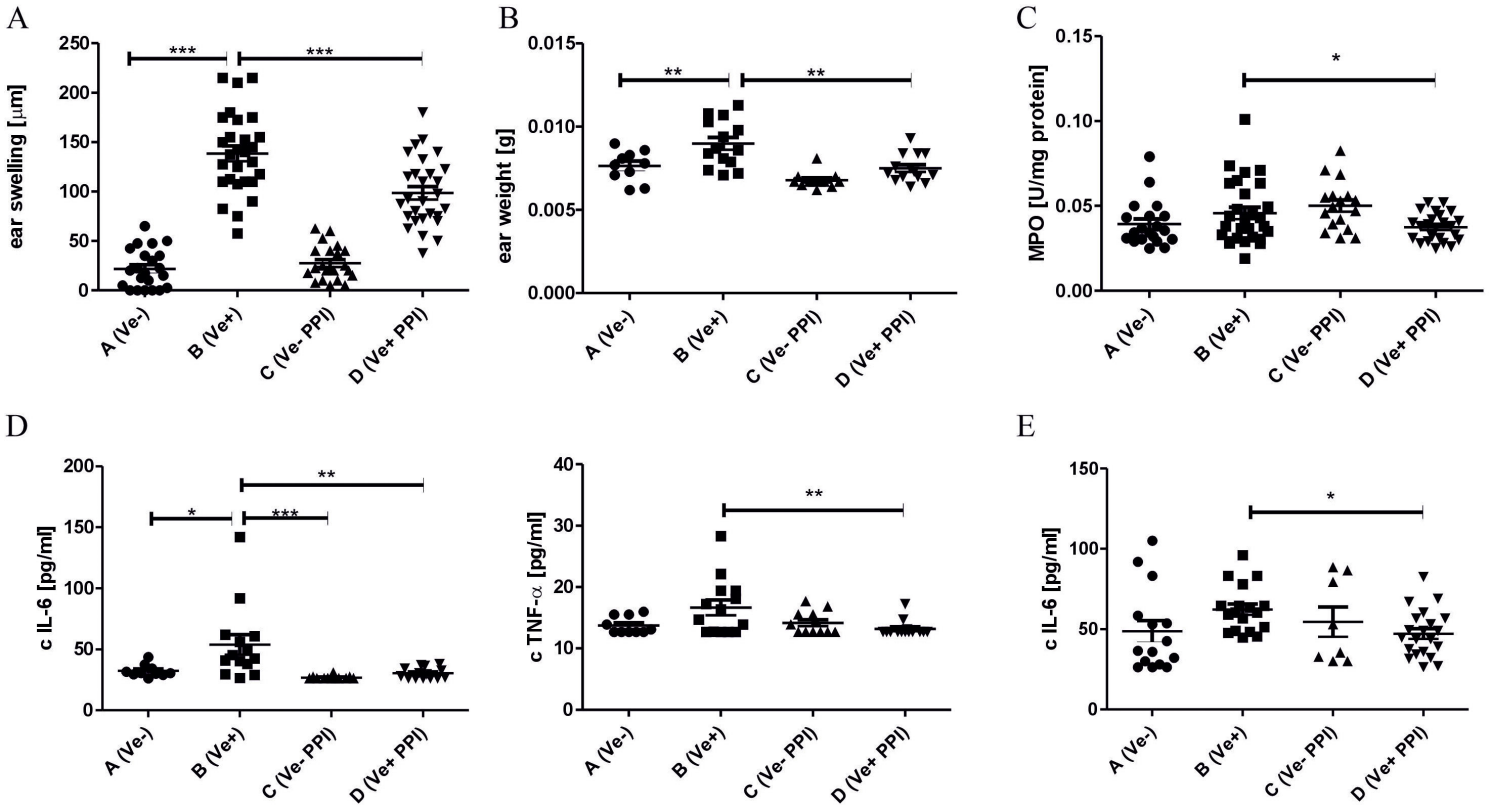
Administration of proton pump inhibitor alleviates CHS in mice. Mice were treated *i.p.* with 15 mg/kg omeprazole in DPBS (Groups C and D) or DPBS alone (Groups A and B) twice daily for 14 days prior to TNP-CL immunization (Groups B and D) and then for the following 4 days until CHS test. The CHS response **(A)**; ear biopsies weight **(B)**; MPO activity in ear biopsies **(C)**; IL-6, TNF-α concentration in ear biopsies **(D)**; IL-6 concentration in ELNC culture supernatants **(E)**. Results are shown as means ± SE. The data presented are combined from two independent experiments **(A–C)** n=20-28; **(D)** n= 8-21; *P < 0.05, and **P < 0.01, and ***P<0.005.

PPIs have been shown to have anti-inflammatory properties, and mice treated with PPIs have altered the composition of gut microbiota (9, 47). The change in the intestinal microbiota profile induced by antibiotic treatment reduced the inflammatory response, leading to an ameliorated CHS reaction (17). To determine the influence of PPIs on CHS, C57BL/6 mice were treated *i.p*. with PPI omeprazole prior to TNP-CL immunization. Data presented in **Figure 1A** show that omeprazole treatment reduces the CHS response measured by ear swelling (Group D vs. B). The ear swelling data correlate with reduced ear biopsy weight (**Figure 1B**; Group D vs. B) and MPO activity (**Figure 1C**; Group D vs. B) in ear biopsy extracts. Furthermore, omeprazole treatment reduces the concentration of proinflammatory IL-6 and TNF-α in ear extracts (**Figure 1D**; Group D vs. B), and IL-6 concentration in supernatants from ELNC culture (**Figure 1E**; Group D vs. B).

### 3.2 Treatment with proton pump inhibitor reduces the inflammatory response in peripheral lymphoid organs

**Figure 2:**
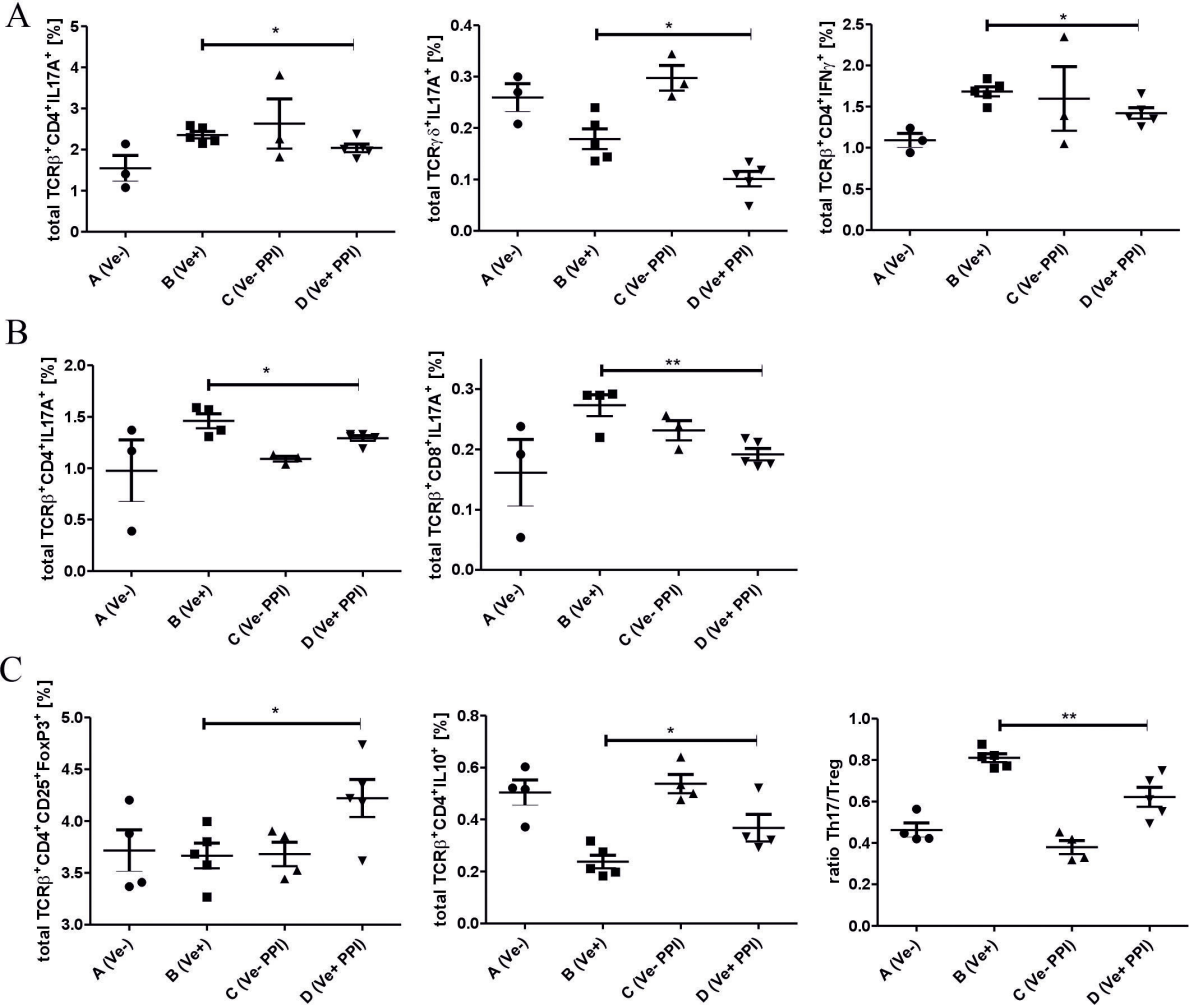
Treatment with a proton pump inhibitor prior to TNP-Cl immunization modulates the inflammatory response in peripheral lymphoid organs. DPBS (Groups A and B) or omeprazole (Groups C and D) treated mice (15 mg/kg in 100ul DPBS) were (Groups B and D) or were not (Groups A and C) sensitized with TNP-Cl prior to FACS analysis of ALNC and SPLC. Percentage of TCRαβ^+^ CD4^+^ IL17A^+^ and TCRγδ^+^ IL17A^+^ and TCRαβ^+^ CD4^+^ IFN-γ^+^ lymphocytes in ALNC **(A)**. Percentage of TCRαβ^+^ CD4^+^ IL17A^+^ and TCRαβ^+^ CD8^+^ IL17A^+^ lymphocytes in SPLC **(B)**. Percentage of TCRαβ^+^ CD4^+^ CD25^+^ FoxP3^+^ Treg, TCRαβ^+^ CD4^+^ IL-10^+^ Tr1 cells, and the ratio between Th17/Treg cells in ALNC **(C)**. Results shown as mean ± SE. Data shown from one representative experiment; n = 3-5. *P < 0.05 and **P < 0.01.

CHS response to TNP-Cl in C57BL/6 is driven by INF-γ- and IL17A-producing CD4^+^ or CD8^+^ T cells (19, 20). To determine the changes in immune response after omeprazole treatment, we phenotyped immune cells in peripheral lymphoid organs by flow cytometry. Our data show that the ameliorated inflammatory response in the skin correlates with a decreased percentage of TCRαβ^+^ CD4^+^ IL17A^+^, TCRγδ^+^ IL17A^+^ and TCRαβ^+^ CD4^+^ IFN-γ^+^ cells in ALNC (**Figure 2A**; Group D vs. B), and TCRαβ^+^ CD4^+^ IL17A^+^ and TCRαβ^+^ CD8^+^ IL17A^+^ cells in SPLC (**Figure 2B** Group D vs. B). Concomitantly, omeprazole treatment prior to TNP-CL immunization increased the frequency of regulatory TCRαβ^+^ CD4^+^ CD25^+^ FoxP3^+^ Treg and TCRαβ^+^ CD4^+^ IL-10^+^ Tr1 cells in ALNC (**Figure 2C**; Group D vs. B), resulting in a decreased ratio between Th17 and Treg cells (**Figure 2C**; Group D vs. B). Our data shows that omeprazole application prior to immunization reduces the frequency of proinflammatory IL17A and IFN-γ T cells and increases the frequency of regulatory T cells.

### 3.3 Administration of proton pump inhibitor shifts immune status in the intestine and gut-associated lymphoid organs towards anti-inflammatory

**Figure 3:**
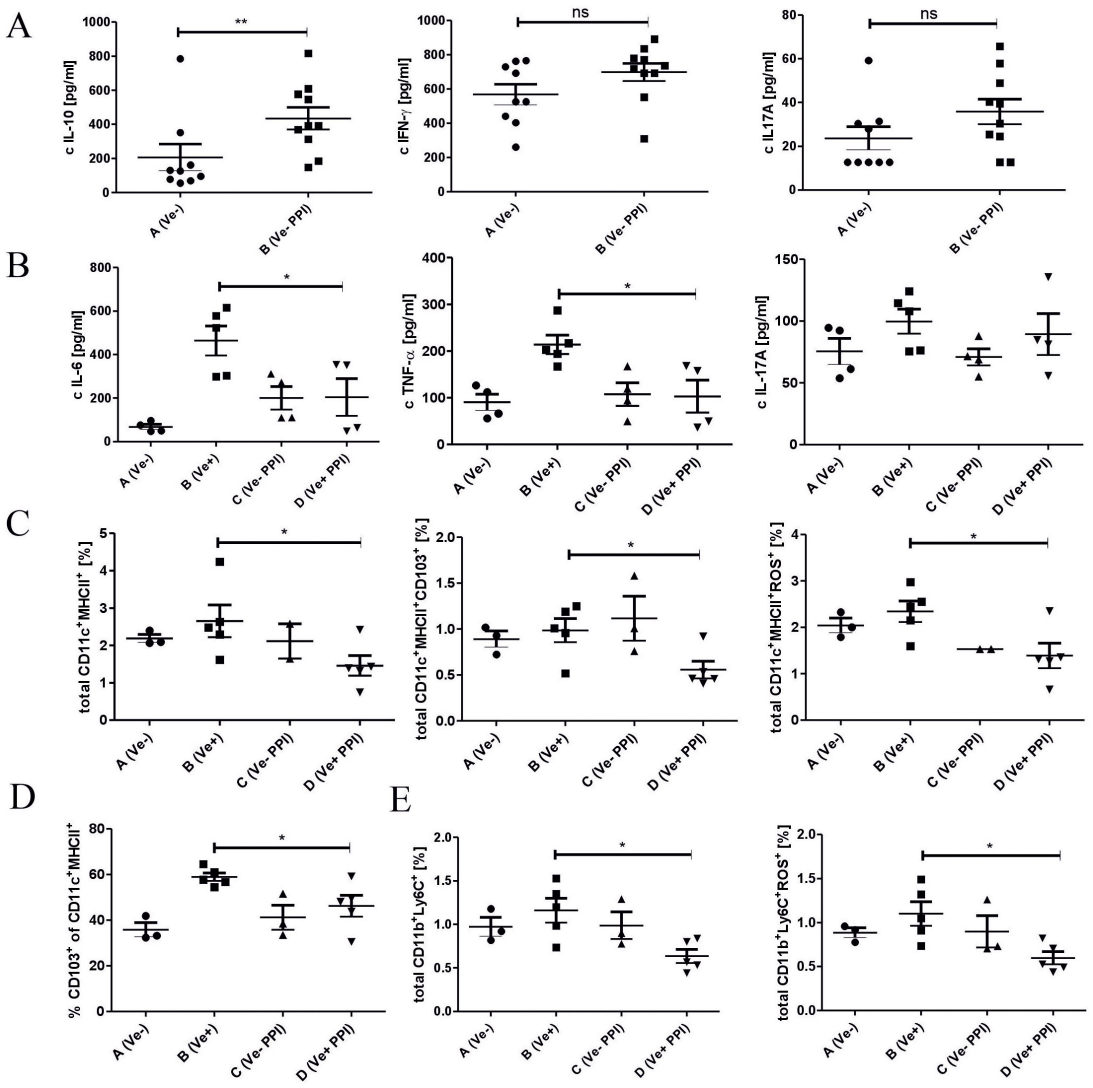
Administration of proton pump inhibitor prior to TNP-Cl immunization induces shifts in immune status in the intestine and gut-associated lymphoid organs towards anti-inflammatory. Mice treated with DPBS or omeprazole (15 mg/kg in 100ul DPBS) were (Groups B and D) or were not (Groups A and C) sensitized with TNP-Cl on shaved abdomen. 4 days later mice were sacrificed, and small intestine tissue biopsies were collected and their homogenates were tested for cytokines using ELISA. Cells isolated from MLN and PP were stained with monoclonal antibodies conjugated with fluorochromes and analyzed using flow cytometry. MLNC were cultured for 2 days with TNP-Ig. Tissue culture supernatants were tested for cytokines using ELISA. The homogenates of small intestine biopsies were tested for IL-10, IFN-γ, and IL-17A **(A)**; MLNC culture supernatants were tested for IL-6, TNF-α, IL17A **(B)**; Percentage of CD11c^+^ MHCII^+^ and CD11c^+^ MHCII^+^ CD103^+^ and CD11c^+^ MHCII^+^ ROS^+^ in MLNC **(C)**; Percentage of CD11c^+^ MHCII^+^ CD103^+^ cells in PP **(D)**; and the percentage of CD11b^+^ Ly6C^+^ and CD11b^+^ Ly6C^+^ROS^+^ cells in MLNC **(E)**. Results shown as mean ± SE. Data shown are from one representative experiment; n = 3-5. * P < 0.05 and ** P < 0.01.

To decipher the basis of the suppression of inflammatory response induced by omeprazole, we evaluated the immune status in the lymphoid organs associated with the intestine. Omeprazole treatment increases the concentration of anti-inflammatory IL-10 and does not affect the concentration of pro-inflammatory IL-17A and IFN-γ in small intestine biopsies (**Figure 3A**; Group B vs. A). Treatment with omeprazole before immunization decreased the concentration of pro-inflammatory IL-6 and TNF-α in culture supernatants of MLN cells (**Figure 3B**; Group D vs B). MLNC flow cytometry analysis revealed that omeprazole treatment prior to immunization with TNP-Cl reduced the frequency of immune cells involved in the induction of T cell-dependent immune response, that is CD11c^+^ MHCII^+^DC, CD11c^+^ MHCII^+^ROS^+^DC, as well as the frequency of migratory CD11c^+^ MHCII^+^ CD103^+^ DC (**Figure 3C**; Group D vs. B). A decreased percentage of CD11c^+^ MHCII^+^ CD103^+^ DC was also observed in PP (**Figure 3D**; Group D vs. B). Furthermore, the frequency of CD11b^+^ Ly6C^+^ and CD11b^+^ Ly6C^+^ROS^+^ monocytes/macrophages (**Figure 3E**; Group D vs. B), was also decreased.

### 3.4 Dysbiosis induced by treatment with proton pump inhibitor modulates CHS response

**Figure 4:**
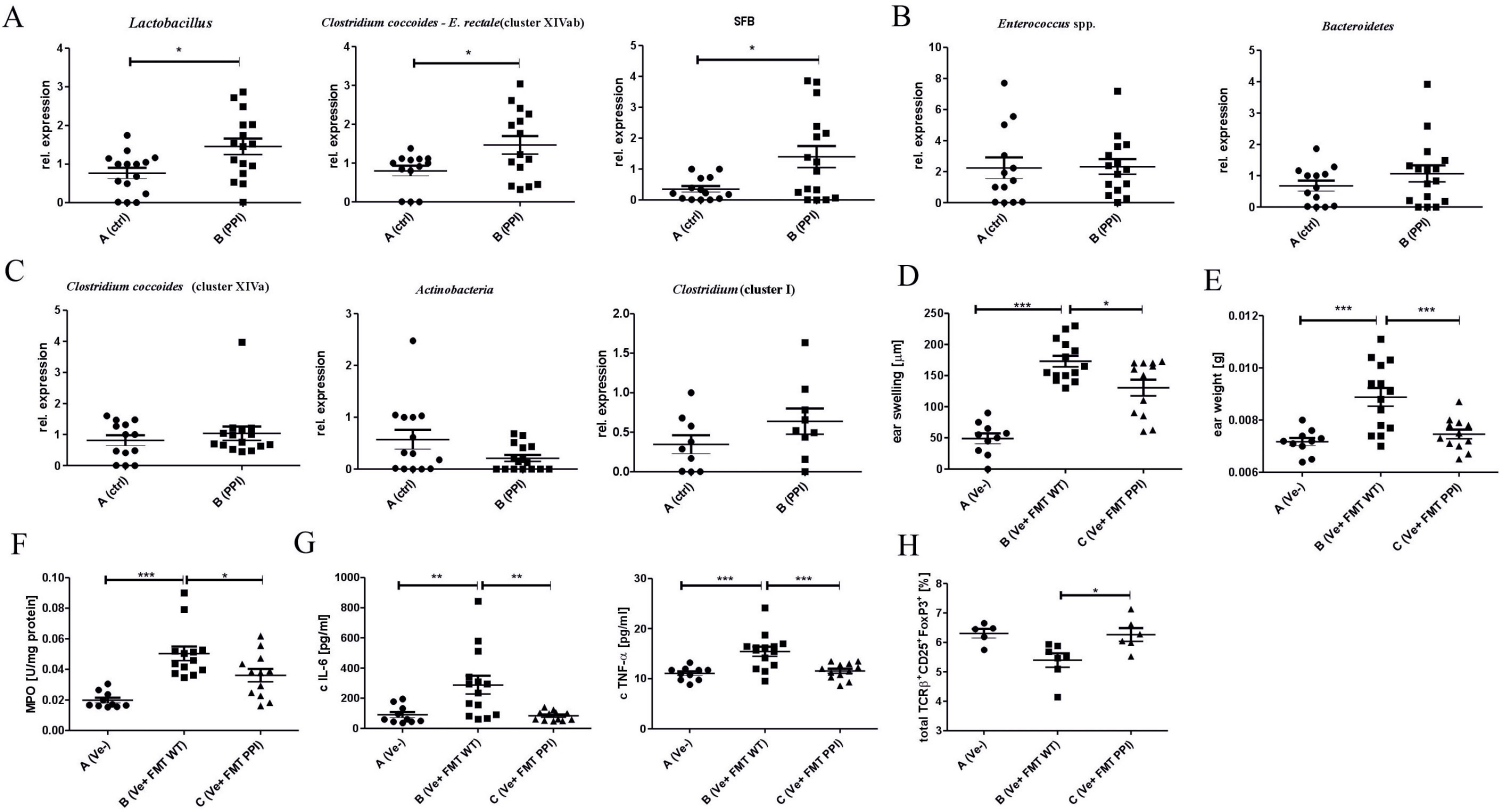
Treatment with proton pump inhibitor influences gut bacteria composition leading to modulated CHS response *in vivo*. Mice were treated with DPBS (Group A) or omeprazole (Group B, 15 mg/kg in 100ul DPBS). Large intestine content was collected and a relative abundance of bacterial conservative 16S rDNA fragments in the gut was evaluated using qPCR: *Lactobacillus, Clostridium coccoides – E. rectale* (cluster XIVab) and SFB **(A)**; *Enterococcus* spp., *Bacteroidetes* **(B)** *Clostridium coccoides* (cluster XIVa), *Actinobacteria* and *Clostridium* cluster I **(C)**. Naïve mice received fecal microbiota transplantation from proton pump (Group C) and DPBS treated (Group B) donor mice, followed by CHS induction and CHS measurement **(D)**. The ear tissue biopsies were collected and weighed **(E)**. The biopsy homogenates were tested for MPO activity **(F)**, and IL-6 and TNF-α concentration **(G**). The ear lymph nodes were collected, stained with monoclonal antibodies conjugated with fluorochromes, and analyzed using flow cytometry **(H)**. Results shown as mean ± SE. Data shown are from one representative experiment; n = 9-16 **(A–C)**, n = 10-14 **(D–H)**; n= 5-7 **(H)** *P < 0.05, **P < 0.01, ***P < 0.005.

Treatment with PPIs is known to influence the composition of the gut microbiota, which is a powerful modulator of the immune response in the intestine and periphery. To test whether an alteration in immune status could be associated with changes in the gut microbiota induced by 2-week omeprazole treatment, we analyzed the composition of some common gut microbiota using RT-PCR. Among the tested species, the relative abundance of *Lactobacillus*, *Clostridium coccoides* – *E. rectale* (cluster XIVab) and SFB was significantly increased in omeprazole-treated mice compared to untreated animals (**Figure 4A**, Group B vs. A), while the relative abundance of *Enterococcus* spp., *Bacteroidetes*, (**Figure 4B**, Group B vs. A), as well *Clostridium coccoides* (cluster XIVa), *Actinobacteria* and *Clostridium* cluster I (**Figure 4C**, Group B vs. A) was similar between untreated and omeprazole-treated mice.

To establish whether the changes in gut microbiota composition result in altered immune response after 2-week omeprazole treatment, the FMT experiment was performed. The transfer of intestinal bacteria from donor mice treated with omeprazole for two weeks (group C; Ve+ FMT PPI) resulted in a decreased inflammatory response in the CHS model compared to the group that received the transfer of control microbiota obtained from donors treated with DPBS (group B; Ve+ FMT WT) (**Figure 4D**, Group C vs. B). The reduced ear swelling response in the FMT group receiving intestinal bacteria from omeprazole-treated donors correlates with decreased ear biopsy weight (**Figure 4E**, Group C vs. B), MPO activity (**Figure 4F**, Group C vs. B), and IL-6 and TNF-α concentrations (**Figure 4G**, Group C vs. B) in ear biopsies. Interestingly, recipients receiving FMT from omeprazole-treated donors show an increased frequency of CD25+ FoxP3+ Treg cells (**Figure 4H**, Group C vs. B) compared to recipients of control microbiota from DPBS-treated donors.

## 4 Discussion

The contact hypersensitivity response (CHS) is a mouse model of allergic contact dermatitis observed in humans, arising after exposure to contact allergens. These low-molecular-weight substances, also known as haptens, include chemicals widely used by society, such as metal ions, dyes, preservatives, and fragrances (23). An estimated 20% of the general population suffers from contact allergy (24). The CHS reaction is mediated by CD4^+^T cells (25) producing IL-17A and IFN-γ (19) and is classified as a type IV hypersensitivity reaction, which characterizes many autoimmune disorders.

Omeprazole is one of the most commonly prescribed drugs (1), and its use could be lifelong. It blocks gastric acid production, leading to changes in intestinal microbiota composition (48). Additionally, it shows anti-inflammatory properties as it regulates ROS and pro-inflammatory cytokine production and suppresses immune cell chemotaxis (10, 13). Since exposure to contact sensitizers and omeprazole is very common, and omeprazole presents immune modulatory properties, this study aimed to evaluate the impact of omeprazole treatment on the development of T cell-dependent immune response using the CHS model.

Previous studies showed that CHS reaction is influenced by a plethora of factors such as antibiotics (17), diet (51), antidepressant drugs (52), which also acts through modulation of intestinal microbiota. In this study, we evaluated the impact of omeprazole on the development of CHS response. Our data indicate that two-week treatment with omeprazole before induction of CHS response reduces the severity of inflammatory response measured by ear swelling (**Figure 1A**), which is confirmed by reduced ear biopsy weight (**Figure 1B**) (26).

Ear swelling 24 h after subsequent exposure to hapten drives infiltration of antigen-specific T lymphocytes and innate immune cells such as MPO-positive neutrophils (26). Our data shows that omeprazole treatment leads to reduced MPO activity in ear biopsies (**Figure 1C**). Additionally, reduced neutrophil infiltration could, in turn, diminish the magnitude of CHS response, as both neutrophils and MPO are implicated in CHS elicitation. Mice depleted of neutrophils transferred with CHS T-effector cells fail to elicit an ear swelling response (27), while MPO deficiency reduces vascular permeability (28).

Reduced CHS response was accompanied by reduced concentration of proinflammatory IL-6 and TNF-α in ear extracts (**Figure 1D**) and IL-6 in ELN cell culture (**Figure 1E**). Both IL-6 and TNF-α could be classified as innate immune cytokines, which are produced by keratinocytes directly stimulated with haptens, or by skin residing innate immune cells stimulated by danger associated molecular patterns (DAMPs) released after hapten application (29). Both cytokines positively regulate CHS response, as IL-6 knock-out and TNF-α knock-out mice have ameliorated ear swelling response (30, 31). Keratinocytes, Langerhans cells, macrophages, or innate lymphoid cells 1 (ILC1) are known sources of TNF-α, which is implicated in T cell infiltration during elicitation by induction of IFN-γ-inducible protein 10 (IP-10, CXCL10) (29, 30). Keratinocytes, Langerhans cells, and macrophages produce IL-6, supporting lymphocyte proliferation in lymph nodes (31).

After painting with hapten, skin resident Langerhans cells pick up antigens and migrate to local draining lymph nodes where the hapten-specific T cells are induced. CHS-mediating T cells belong to the CD8^+^ or CD4^+^ population and produce IL-17A or IFN-γ (19) as knock-out mice of IL-17A and IFN-γ (32), and mice deficient in receptors for IL-17A, or IFN-γ have reduced CHS response (19). Subcutaneous injection of recombinant IL-17A or IFN-γ enhanced CHS manifestation, while depletion using mAb of IFN-γ in IL-17 R^−/−^ mice and depletion of IL-17A in IFN-γ R^−/−^ mice further suppressed the CHS response compared to IL-17 R^−/−^ or IFN-γ R^−/−^ (19). Our data shows that omeprazole application prior to immunization results in a reduced percentage of TCRαβ^+^ CD4^+^ IFN-γ^+^ and TCRαβ^+^ CD4^+^ IL-17A^+^ lymphocytes in ALNC (**Figure 2A**) and TCRαβ^+^ CD4^+^ IL-17A^+^ and TCRαβ^+^ CD8^+^ IL-17A^+^ lymphocytes in SPLC (**Figure 2B**), suggesting that the application of omeprazole interferes with the induction of antigen-specific T cells. Furthermore, the development of CHS response is supported by γδ^+^T cells, as adoptive transfer of LN cells depleted of γδ^+^T reduces the ear swelling response (33, 48). The γδ^+^T cells are a known source of IL-17A, but could also promote the CHS response by supporting IFN-γ, IL-12, and TNF-α production by LN cells (33). In agreement with the role of γδ^+^T cells, we observed that reduced CHS response after omeprazole application is associated with a lower frequency of TCRγδ^+^ IL-17A^+^ lymphocytes in ALNC (**Figure 2A**).

Although the depletion studies revealed that CHS-effector T cells belong to CD8^+^ and CD4^+^ populations, they also showed that some fraction of CD4^+^ could have regulatory properties (25). Further research revealed that CHS response is negatively regulated by TCRαβ^+^ CD4^+^ CD25^+^ FoxP3^+^ Treg cells, CD19^+^ B220^+^ CD5^+^ IL-10^+^ Breg cells, IL-10^+^ Tr1, or IL-10^+^ TCR γδ^+^ lymhocytes (17, 34). FACS analysis of LN cells from omeprazole-treated mice showed an increased number of CD4^+^ CD25^+^ FoxP3^+^ Treg, and CD4^+^ IL-10^+^ Tr1 cells, suggesting that omeprazole application favors the development of T regulatory cells (**Figure 2C**). Consistent with our results, omeprazole treatment increases the frequency of CD4^+^ CD25^+^ FoxP3^+^ Treg cells and decreases the frequency of CD4^+^ IL-17A^+^ T cells among peripheral blood mononuclear cells (PBMCs) of patients with duodenal ulcers. The shift was accompanied by a reduced concentration of IL-17A and an increased concentration of IL-10 (35). Treg cells restrict inflammatory response by preventing the induction of antigen-specific cells and limiting their proliferation. Consistently, our data shows a reduced frequency of IFN-γ^+^- and IL-17A^+^ -producing T cells.

To further understand immune changes induced by omeprazole application, we evaluated the immune status in the intestine and gut-associated lymphoid organs. Previous studies showed that reduced CHS response due to enrofloxacin-induced dysbiosis was associated with the anti-inflammatory milieu in the intestinal tissue, characterized by increased production of IL-10 and no change in the level of IFN-γ and IL-17A (50). Similarly, a two-week omeprazole application leading to reduced CHS, increased the concentration of anti-inflammatory IL-10 and had no impact on pro-inflammatory IL-17A and IFN-γ concentrations in small intestine biopsies (**Figure 3A**). The increase in IL-10 production after omeprazole application is consistent with study showing that patients with gastric ulcer treated with omeprazole combined with antibiotic mixture have increased levels of IL-10 in serum and in the ulcer tissue compared to patients treated with bismuth subcitrate combined with antibiotic mixture (35). IL-10 was implicated in the suppression of ROS production pro-inflammatory IL-1β, IL-6, and TNF-α, and suppression of adaptive immune response induction by inhibiting antigen presentation through the reduction of MHC II and costimulatory molecule expression (50). Consistently, we observed reduced production of IL-6 and TNF-α by MLN cells (**Figure 3B**). Additionally, the reduced production of IL-6 and TNF-α could also be the consequence of the direct influence of omeprazole on immune cells, as the studies evaluating the impact of PPIs on immune response, showed decreased production of TNF-α and tendency towards reduced production of IL-6 by THP-1 cell line exposed to Lansoprazole (10). However, another study reported that two-weeks omeprazole application restored to the control level the production of IL-1β, IL-6, and TNF-α in nerve tissue homogenate from chronic constriction injury (CCI)-induced rat model of neuropathic pain (11).

The cellular sources of IL-6 and TNF-α are dendritic cells and macrophages (29). In our study, we also observed that omeprazole application reduced the frequency of DC CD11c^+^ MHCII^+^, migratory DC CD11c^+^ MHCII^+^ CD103^+^ (**Figure 3C**), and CD11b^+^ Ly6C^+^ monocytes/macrophages (**Figure 3E**). Both cytokines play a crucial role in the development of Th17 cells, as IL-6 prime the development of Th17 cells while the influence of TNF-α is indirect, as it promotes production of IL-1β and IL-6 from monocytes (36). CD11c^+^ MHCII+ CD103^+^ DCs are the principal fraction of migratory DC in MLNC and are known sources of IL-6 required for induction of Th17-dependent immune response (37). Additionally, CD103+ DCs, but not CD103− DCs, support the induction of CD4+CD25+FoxP3+ Treg cells. Mice lacking CD103+ antigen-presenting cells were unable to restrict T cell-mediated colitis (38, 39). The migration of CD11c+ MHCII+ CD103+ DCs to the MLN is driven by chemokines (40), which are also produced by the lymph nodes and spleen, suggesting the potential for CD103+ DCs to migrate to peripheral tissues, where they could support the induction of Treg cells (41).

Additionally, we found that omeprazole application reduced the production of ROS, as we observed reduced frequency of CD11c^+^ MHCII^+^ ROS^+^ DC (**Figure 3C**) and CD11b^+^ Ly6C^+^ROS^+^ monocytes/macrophages (**Figure 3E**). This is consistent with the studies showing that omeprazole application increase the level of antioxidant superoxide dismutase (SOD), and concomitantly the level of reduced glutathione (GSH) (11). ROS produced by DC supports the induction of adaptive immune response, and inhibition of redox-sensitive pathways prevents the induction of antigen-specific T cells in the CHS model (21). Thus, reduced level of ROS, along with reduced frequency of CD11c^+^ MHCII^+^ CD103^+^ DC and lower production of IL-6 and TNF-α after omeprazole treatment, could account for impaired induction of TCRαβ^+^ CD4^+^ IL-17A^+^ and TCRαβ^+^ CD8^+^ IL-17A^+^ T cells (**Figure 2A** and **Figure 2B**), leading to reduced CHS response.

Dysbiosis is a change in bacterial composition, due to loss of beneficial bacteria, overgrowth of potentially pathogenic ones, or decrease in bacterial diversity or distribution within intestines (42). The application of PPIs changes the composition of intestinal microbiota (47). Gut microbiota is an established modulator of peripheral immune response, including the CHS model (17). Our data shows that a two-week omeprazole treatment changes intestinal microbiota composition. However, we did not observe changes in the relative abundance of *Enterococcus* spp.*, Bacteroidetes* (**Figure 4B**), as well *Clostridium coccoides* (cluster XIVa)*, Actinobacteria* and *Clostridium* cluster I (**Figure 4C**); omeprazole application increases the abundance of *Lactobacillus, Clostridium coccoides* – *E. rectale* (cluster XIVab) and *SFB* (**Figure 4A**). Although the impact of omeprazole on gut microbiota is undisputable, the exact changes could vary depending on dosing regimen, and tested model (15, 48).

Consistently to our study, a 4-week omeprazole treatment caused a major increase in *Lactobacillus* levels (43). *Lactobacillus* strains are well-known probiotics with proven anti-inflammatory action. Supplementation with *Lactobacillus johnsonii* efficiently reduced DSS-induced colitis, which was associated with decreased levels of TNF-α and IL-6 and increased levels of IL-10 and IL-17 (44). Additionally, bacteria belonging to *Clostridium cluster* XIVa support induction of FoxP3-expressing T cells (45), and the increase in their abundance after antibiotic treatment was associated with reduced CHS response (17). The role of omeprazole-induced dysbiosis in the regulation of immune response in the CHS model was confirmed using FMT (**Figure 4D**). The decreased ear swelling response correlated with a reduction in ear biopsy weight (**Figure 4E**), MPO activity (**Figure 4F**), and the concentrations of IL-6 and TNF-α (**Figure 4G**). These changes were observed following a two-week omeprazole treatment administered prior to immunization and challenge (**Figures 1A–D**). Additionally, flow cytometry analysis of ELN revealed an increased frequency of TCRαβ+ CD25+ FoxP3+ regulatory cells (**Figure 4H**), which, contribute to maintaining tolerance (46). Our data suggest that at least partially that the ameliorated CHS response after omeprazole treatment is the consequence of gut microbiota changes.

In conclusion, a two-week omeprazole treatment reduces CHS response by shifting immune status towards anti-inflammatory, characterized by increased IL-10 and decreased IL-6 and TNF-α production, which could be the consequence of the direct impact of omeprazole on immune cells or alteration of gut microbiota composition. The changes in immune cells and microbiota profiles after omeprazole application support the development of Treg cells while preventing the induction of Th17 lymphocytes. Alteration of Th17/Treg cells balance observed after omeprazole treatment suppresses CHS response.

## Data Availability

The raw data supporting the conclusions of this article will be made available by the authors, without undue reservation.

## Supplementary material

Scheme 1 Scheme of the experiment.

## References

[1] Strand DS Kim D Peura DA. 25 years of proton pump inhibitors: A comprehensive review. Gut Liver. (2017) 11:27–37. doi: 10.5009/gnl15502 27840364 PMC5221858

[2] Haastrup PF Paulsen MS Christensen RD Søndergaard J Hansen JM Jarbøl DE. Medical and non-medical predictors of initiating long-term use of proton pump inhibitors: a nationwide cohort study of first-time users during a 10-year period. Aliment Pharmacol Ther. (2016) 44:78–87. doi: 10.1111/apt.13649 27137875

[3] Vich Vila A Collij V Sanna S Sinha T Imhann F Bourgonje AR. Impact of commonly used drugs on the composition and metabolic function of the gut microbiota. Nat Commun. (2020) 11:362. doi: 10.1038/s41467-019-14177-z 31953381 PMC6969170

[4] Sheen E Triadafilopoulos G. Adverse effects of long-term proton pump inhibitor therapy. Dig Dis Sci. (2011) 56:931–50. doi: 10.1007/s10620-010-1560-3 21365243

[5] Clooney AG Bernstein CN Leslie WD Vagianos K Sargent M Laserna-Mendieta EJ. A comparison of the gut microbiome between long-term users and non-users of proton pump inhibitors. Aliment Pharmacol Ther. (2016) 43:974–84. doi: 10.1111/apt.13568 26923470

[6] Epstein M McGrath S Law F. Proton-pump inhibitors and hypomagnesemic hypoparathyroidism. N Engl J Med. (2006) 355:1834–6. doi: 10.1056/NEJMc066308 17065651

[7] Vesper BJ Jawdi A Altman KW Haines GK 3rd Tao L Radosevich JA. The effect of proton pump inhibitors on the human microbiota. Curr Drug Metab. (2009) 10:84–9. doi: 10.2174/138920009787048392 19149516

[8] Tian L Huang C Fu W Gao L Mi N Bai M. Proton pump inhibitors may enhance the risk of digestive diseases by regulating intestinal microbiota. Front Pharmacol. (2023) 14:1217306. doi: 10.3389/fphar.2023.1217306 37529701 PMC10387554

[9] Biswas S Benedict SH Lynch SG LeVine SM. Potential immunological consequences of pharmacological suppression of gastric acid production in patients with multiple sclerosis. BMC Med. (2012) 10:57. doi: 10.1186/1741-7015-10-57 22676575 PMC3386885

[10] Tanigawa T Watanabe T Higuchi K Machida H Okazaki H Yamagami H. Lansoprazole, a proton pump inhibitor, suppresses production of tumor necrosis factor-alpha and interleukin-1beta induced by lipopolysaccharide and helicobacter pylori bacterial components in human monocytic cells via inhibition of activation of nuclear factor-kappaB and extracellular signal-regulated kinase. J Clin Biochem Nutr. (2009) 45:86–92. doi: 10.3164/jcbn.08-267 19590712 PMC2704330

[11] Chanchal SK Mahajan UB Siddharth S Reddy N Goyal SN Patil PH. *In vivo* and in *vitro* protective effects of omeprazole against neuropathic pain. Sci Rep. (2016) 6:30007. doi: 10.1038/srep30007 27435304 PMC4951708

[12] Cortes JR Rivas MD Molina-Infante J Gonzalez-Nuñez MA Perez-G M Masa JF. Omeprazole inhibits IL-4 and IL-13 signaling signal transducer and activator of transcription 6 activation and reduces lung inflammation in murine asthma. J Allergy Clin Immunol. (2009) 124:607–10, 610.e1. doi: 10.1016/j.jaci.2009.06.023 19665777

[13] Wandall JH. Effects of omeprazole on neutrophil chemotaxis, super oxide production, degranulation, and translocation of cytochrome b-245. Gut. (1992) 33:617–21. doi: 10.1136/gut.33.5.617 PMC1379289 1319381

[14] Ohara T Arakawa T. Lansoprazole decreases peripheral blood monocytes and intercellular adhesion molecule-1-positive mononuclear cells. Dig Dis Sci. (1999) 44:1710–5. doi: 10.1023/a:1026604203237 10492157

[15] Sands SA Tsau S Yankee TM Parker BL Ericsson AC LeVine SM. The effect of omeprazole on the development of experimental autoimmune encephalomyelitis in C57BL/6J and SJL/J mice. BMC Res Notes. (2014) 7:605. doi: 10.1186/1756-0500-7-605 25190469 PMC4167283

[16] Martin SF. Immunological mechanisms in allergic contact dermatitis. Curr Opin Allergy Clin Immunol. (2015) 15:124–30. doi: 10.1097/ACI.0000000000000142 25611878

[17] Strzępa A Majewska-Szczepanik M Lobo FM Wen L Szczepanik M. Broad spectrum antibiotic enrofloxacin modulates contact sensitivity through gut microbiota in a murine model. J Allergy Clin Immunol. (2017) 140:121–33. doi: 10.1016/j.jaci.2016.11.052 28130148

[18] Enk AH Katz SI. Contact sensitivity as a model for T-cell activation in skin. J Invest Dermatol. (1995) 105:80S–3S. doi: 10.1111/1523-1747.ep12316112 7616003

[19] He D Wu L Kim HK Li H Elmets CA Xu H. IL-17 and IFN-gamma mediate the elicitation of contact hypersensitivity responses by different mechanisms and both are required for optimal responses. J Immunol. (2009) 183:1463–70. doi: 10.4049/jimmunol.0804108 PMC3179907 19553527

[20] Kish DD Li X Fairchild RL. CD8 T cells producing IL-17 and IFN-gamma initiate the innate immune response required for responses to antigen skin challenge. J Immunol. (2009) 182:5949–59. doi: 10.4049/jimmunol.0802830 PMC2860745 19414746

[21] Matsue H Edelbaum D Shalhevet D Mizumoto N Yang C Mummert ME. Generation and function of reactive oxygen species in dendritic cells during antigen presentation. J Immunol. (2003) 171:3010–8. doi: 10.4049/jimmunol.171.6.3010 12960326

[22] de Winter JCF. Using the Student’s t-test with extremely small sample sizes. Pract Assess Res Eval. (2013) 18:1–12.

[23] Johansen JD Bonefeld CM Schwensen JFB Thyssen JP Uter W. Novel insights into contact dermatitis. J Allergy Clin Immunol. (2022) 149:1162–71. doi: 10.1016/j.jaci.2022.02.002 35183605

[24] Alinaghi F Bennike NH Egeberg A Thyssen JP Johansen JD. Prevalence of contact allergy in the general population: A systematic review and meta-analysis. Contact Dermatitis. (2019) 80:77–85. doi: 10.1111/cod.13119 30370565

[25] Gocinski BL Tigelaar RE. Roles of CD4+ and CD8+ T cells in murine contact sensitivity revealed by in *vivo* monoclonal antibody depletion. J Immunol. (1990) 144:4121–8. doi: 10.4049/jimmunol.144.11.4121 1971294

[26] Zemelka-Wiącek M Majewska-Szczepanik M Pyrczak W Szczepanik M. Complementary methods for contact hypersensitivity (CHS) evaluation in mice. J Immunol Methods. (2013) 387:270–5. doi: 10.1016/j.jim.2012.11.004 23183274

[27] Weber FC Nemeth T Csepregi JZ Dudeck A Roers A Ozsvari B. Neutrophils are required for both the sensitization and elicitation phase of contact hypersensitivity. J Exp Med. (2015) 212:15–22. doi: 10.1084/jem.20130062 25512469 PMC4291534

[28] Strzepa A Gurski CJ Dittel LJ Szczepanik M Pritchard KA Jr Dittel BN. Neutrophil-derived myeloperoxidase facilitates both the induction and elicitation phases of contact hypersensitivity. Front Immunol. (2021) 11:608871. doi: 10.3389/fimmu.2020.608871 33569056 PMC7868335

[29] Yamaguchi HL Yamaguchi Y Peeva E. Role of innate immunity in allergic contact dermatitis: an update. Int J Mol Sci. (2023) 24:12975. doi: 10.3390/ijms241612975 37629154 PMC10455292

[30] Nakae S Komiyama Y Narumi S Sudo K Horai R Tagawa Y. IL-1-induced tumor necrosis factor-alpha elicits inflammatory cell infiltration in the skin by inducing IFN-gamma-inducible protein 10 in the elicitation phase of the contact hypersensitivity response. Int Immunol. (2003) 15:251–60. doi: 10.1093/intimm/dxg028 12578855

[31] Hope JC Campbell F Hopkins SJ. Deficiency of IL-2 or IL-6 reduces lymphocyte proliferation, but only IL-6 deficiency decreases the contact hypersensitivity response. Eur J Immunol. (2000) 30:197–203. doi: 10.1002/1521-4141(200001)30:1<197::AID-IMMU197>3.0.CO;2-9 10602041

[32] Nakae S Komiyama Y Nambu A Sudo K Iwase M Homma I. Antigen-specific T cell sensitization is impaired in IL-17-deficient mice, causing suppression of allergic cellular and humoral responses. Immunity. (2002) 17:375–87. doi: 10.1016/S1074-7613(02)00391-6 12354389

[33] Strzepa A Majewska-Szczepanik M Szczepanik M. GammadeltaT cells positively regulate contact sensitivity (CS) reaction via modulation of INF-gamma, IL-12 and TNF-alpha production. Folia Biol (Krakow). (2013) 61:205–10. doi: 10.3409/fb61_3-4.205 24279170

[34] Zemelka-Wiącek M Majewska-Szczepanik M Ptak W Szczepanik M. Epicutaneous immunization with protein antigen induces antigen-non-specific suppression of CD8 T cell mediated contact sensitivity. Pharmacol Rep. (2012) 64:1485–96. doi: 10.1016/s1734-1140(12)70946-5 23406759

[35] Li CY Wu C. Therapy with omeprazole modulates regulatory T cell/T helper 17 immune response in children with duodenal ulcers. Inflammopharmacology. (2018) 26:337–47. doi: 10.1007/s10787-017-0380-x 28735449

[36] Zheng Y Sun L Jiang T Zhang D He D Nie H. TNFα promotes Th17 cell differentiation through IL-6 and IL-1β produced by monocytes in rheumatoid arthritis. J Immunol Res. (2014) 2014:385352. doi: 10.1155/2014/385352 25436214 PMC4243768

[37] Persson EK Uronen-Hansson H Semmrich M Rivollier A Hagerbrand K Marsal J. IRF4 transcription-factor-dependent CD103^+^CD11b^+^ dendritic cells drive mucosal T helper 17 cell differentiation. Immunity. (2013) 38:958–69. doi: 10.1016/j.immuni.2013.03.009 23664832

[38] Annacker O Coombes JL Malmstrom V Uhlig HH Bourne T Johansson-Lindbom B. Essential role for CD103 in the T cell-mediated regulation of experimental colitis. J Exp Med. (2005) 202:1051–61. doi: 10.1084/jem.20040662 PMC2213206 16216886

[39] Coombes JL Siddiqui KR Arancibia-Cárcamo CV Hall J Sun CM Belkaid Y. A functionally specialized population of mucosal CD103+ DCs induces Foxp3+ regulatory T cells via a TGF-beta and retinoic acid-dependent mechanism. J Exp Med. (2007) 204:1757–64. doi: 10.1084/jem.20070590 PMC2118683 17620361

[40] Worbs T Bode U Yan S Hoffmann MW Hintzen G Bernhardt G. Oral tolerance originates in the intestinal immune system and relies on antigen carriage by dendritic cells. J Exp Med. (2006) 203:519–27. doi: 10.1084/jem.20052016 PMC2118247 16533884

[41] Hong W Yang B He Q Wang J Weng Q. New insights of CCR7 signaling in dendritic cell migration and inflammatory diseases. Front Pharmacol. (2022) 13:841687. doi: 10.3389/fphar.2022.841687 35281921 PMC8914285

[42] DeGruttola AK Low D Mizoguchi A Mizoguchi E. Current understanding of dysbiosis in disease in human and animal models. Inflammation Bowel Dis. (2016) 22:1137–50. doi: 10.1097/MIB.0000000000000750 PMC4838534 27070911

[43] Gommers LMM Ederveen THA van der Wijst J Overmars-Bos C Kortman GAM Boekhorst J. Low gut microbiota diversity and dietary magnesium intake are associated with the development of PPI-induced hypomagnesemia. FASEB J. (2019) 33:11235–46. doi: 10.1096/fj.201900839R 31299175

[44] Yuan L Zhu C Gu F Zhu M Yao J Zhu C. *Lactobacillus johnsonii* N5 from heat stress-resistant pigs improves gut mucosal immunity and barrier in dextran sodium sulfate-induced colitis. Anim Nutr. (2023) 15:210–24. doi: 10.1016/j.aninu.2023.04.012 PMC10685162 38033603

[45] Atarashi K Tanoue T Shima T Imaoka A Kuwahara T Momose Y. Induction of colonic regulatory T cells by indigenous Clostridium species. Science. (2011) 331:337–41. doi: 10.1126/science.1198469 PMC3969237 21205640

[46] Liston A Aloulou M. A fresh look at a neglected regulatory lineage: CD8^+^Foxp3^+^ Regulatory T cells. Immunol Lett. (2022) 247:22–6. doi: 10.1016/j.imlet.2022.05.004 35609830

[47] Imhann F Bonder MJ Vich Vila A Fu J Mujagic Z Vork L. Proton pump inhibitors affect the gut microbiome. Gut. (2016) 65:740–8. doi: 10.1136/gutjnl-2015-310376 PMC4853569 26657899

[48] Szczepanik M Ptak W Askenase PW. Role of interleukin-4 in down-regulation of contact sensitivity by gammadelta T cells from tolerized T-cell receptor alpha-/- mice. Immunology. (1999) 98:63–70. doi: 10.1046/j.1365-2567.1999.00837.x 10469235 PMC2326908

[49] Yoshida N Yoshikawa T Tanaka Y Fujita N Kassai K Naito Y. A new mechanism for anti-inflammatory actions of proton pump inhibitors–inhibitory effects on neutrophil-endothelial cell interactions. Aliment Pharmacol Ther. (2000) 14 Suppl 1:74–81. doi: 10.1046/j.1365-2036.2000.014s1074.x 10807407

[50] Strzępa A Marcińska K Majewska-Szczepanik M Szczepanik M. Oral treatment with enrofloxacin creates anti-inflammatory environment that supports induction of tolerogenic dendritic cells. Int Immunopharmacol. (2019) 77:105966. doi: 10.1016/j.intimp.2019.105966 31679846

[51] Majewska-Szczepanik M Kowalczyk P Marcińska K Strzępa A Lis GJ Wong FS. Obesity aggravates contact hypersensitivity reaction in mice. Contact Dermatitis. (2022) 87(1):28–39. doi: 10.1111/cod.14088 35234303 PMC9949724

[52] Kubera M Curzytek K Majewska-Szczepanik M Szczepanik M Marcińska K Ptak W. Inhibitory effect of antidepressant drugs on contact hypersensitivity reaction. Pharmacol Rep. (2012) 64(3):714–22. doi: 10.1016/s1734-1140(12)70866-6 22814024

