# Supplementary material for: Proton pump inhibitor alters Th17/Treg balance and induces gut dysbiosis suppressing contact hypersensitivity reaction in mice
Source: Front Immunol. 2024 Aug 23;15:1390025. doi: 10.3389/fimmu.2024.1390025 (PMC11377960; doi:10.3389/fimmu.2024.1390025)

Gating strategy

A) TCRαβ^{+} CD4^{+} CD25^{+} FoxP3^{+} Treg

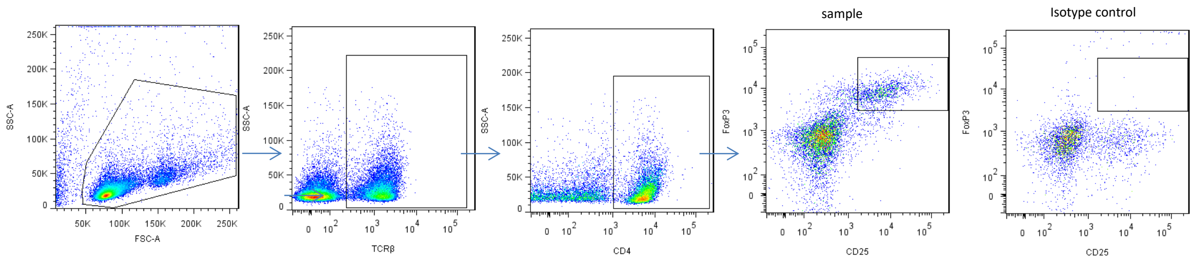

B) TCRαβ^{+} CD4^{+} IFN-γ^{+} and TCRαβ^{+} CD8^{+} IFN-γ^{+}

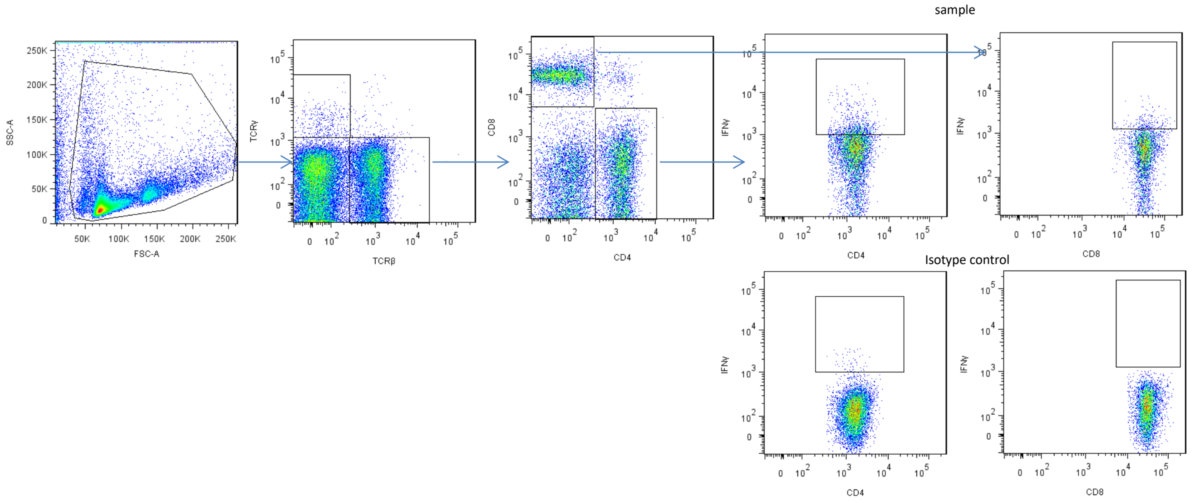

C) TCRαβ^{+} CD4^{+} IL-17A^{+} and TCRαβ^{+} CD8^{+} IL-17A^{+} T cells

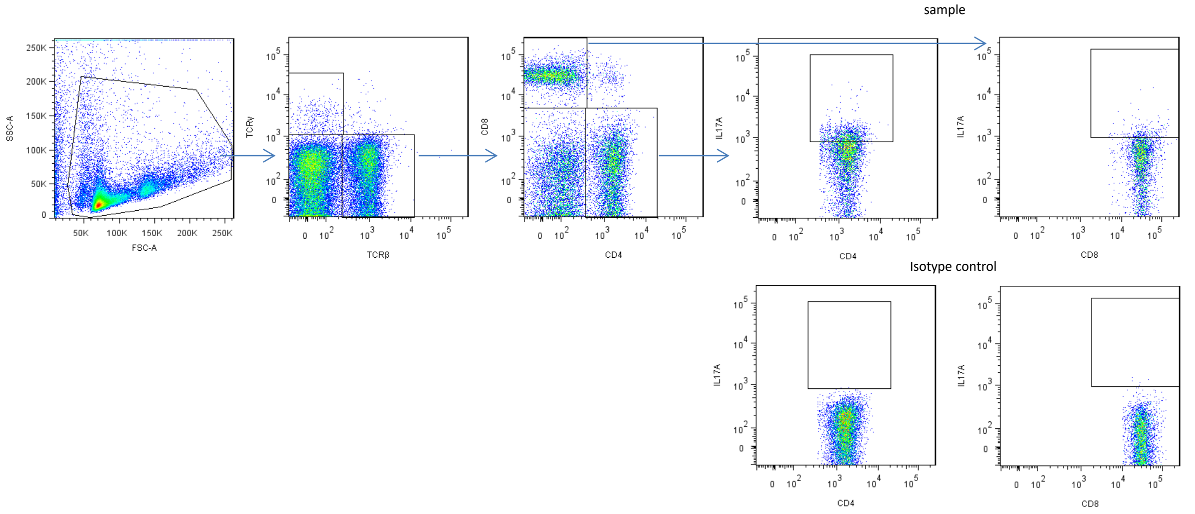

D) TCRαβ^{+} CD4^{+} IL-10^{+} Tr1

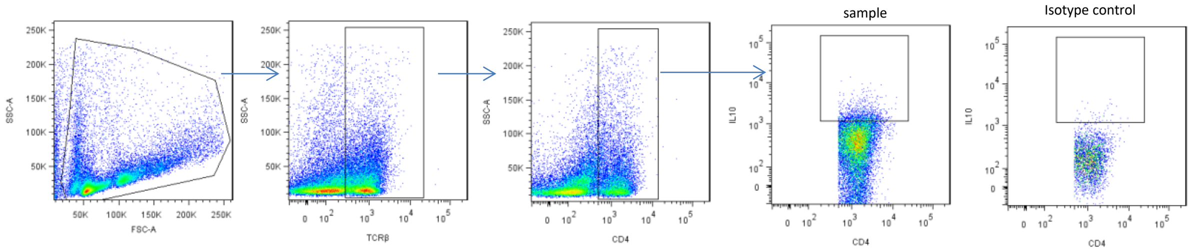

E) CD11b^{+} Ly6C^{+}ROS^{+} monocytes/macrophages

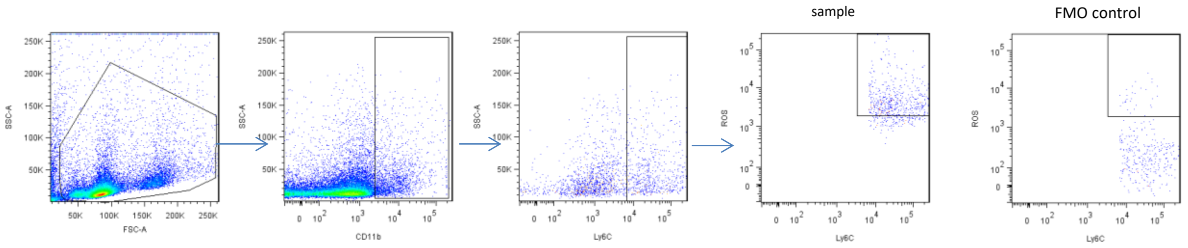

F) CD11c^{+} MHCII^{+}ROS^{+}DC

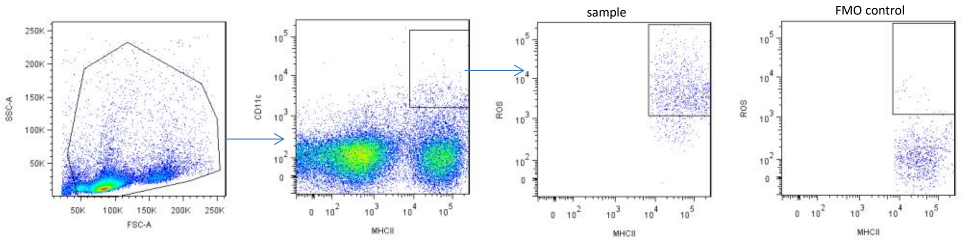

G) CD11c+ MHCII+ CD103+ DC

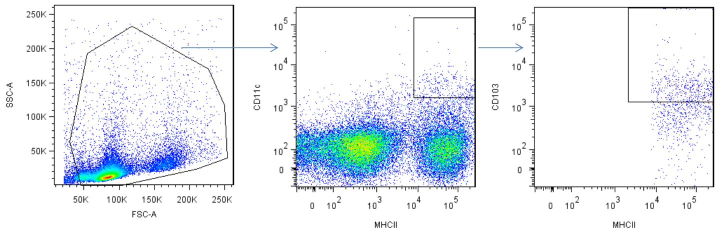

Supplement: Scheme 1 — Scheme of the experiment. [file DataSheet1.pdf]
